# Origin and Diversification of South American Polyploid *Silene* Sect. *Physolychnis* (Caryophyllaceae) in the Andes and Patagonia

**DOI:** 10.3389/fgene.2018.00639

**Published:** 2018-12-11

**Authors:** Božo Frajman, Peter Schönswetter, Hanna Weiss-Schneeweiss, Bengt Oxelman

**Affiliations:** ^1^Department of Botany, University of Innsbruck, Innsbruck, Austria; ^2^Department of Botany and Biodiversity Research, University of Vienna, Vienna, Austria; ^3^Department of Plant and Environmental Sciences, University of Gothenburg, Gothenburg, Sweden

**Keywords:** Andes, chromosome counts, ITS, Patagonia, phylogeny, relative genome size

## Abstract

The Andes are an important biogeographic region in South America extending for about 8000 km from Venezuela to Argentina. They are – along with the Patagonian steppes – the main distribution area of ca. 18 polyploid species of *Silene* sect. *Physolychnis*. Using nuclear ITS and plastid *psbE*-*petG* and *matK* sequences, flow cytometric ploidy level estimations and chromosome counts, and including 13 South American species, we explored the origin and diversification of this group. Our data suggest a single, late Pliocene or early Pleistocene migration of the North American *S. verecunda* lineage to South America, which was followed by dispersal and diversification of this tetraploid lineage in the Andes, other Argentinian mountain ranges and the Patagonian steppes. Later in the Pleistocene South American populations hybridized with the *S. uralensis* lineage, which led to allopolyploidisation and origin of decaploid *S. chilensis* and *S. echegarayi* occurring at high elevations. Additionally, we show that the morphological differentiation in leaf shape correlated with divergent habitats (high elevation Andes vs. lower elevation Patagonian steppes) is also supported phylogenetically, especially in the ITS tree. Lastly, the species boundaries among the narrow-leaved Patagonian steppe species are poorly resolved and need more thorough taxonomic revision.

## Introduction

Polyploidy has significantly contributed to the diversification and radiation of flowering plants (Soltis et al., 2009, 2016; Wood et al., 2009; Husband et al., 2013; Madlung, 2013) and at least two polyploidisation rounds have occurred in all angiosperms (Jiao et al., 2011). Polyploid lineages often exhibit complex relationships among each other and with their lower-ploid ancestors (e.g., Popp et al., 2005; Marhold and Lihova, 2006; Brysting et al., 2011; Frajman et al., 2016). Recurrent formation of polyploids, (epi)genetic, transcriptomic and genomic changes as well as morphological, geographic and ecological divergence following polyploidisation are considered significant processes in the evolution of polyploids (Leitch and Bennett, 2004; Adams and Wendel, 2005; Johnston et al., 2005; Parisod et al., 2010; Sonnleitner et al., 2010; Balao et al., 2011; Weiss-Schneeweiss et al., 2013), and obviously increase taxonomic complexity. On the other hand, polyploids have been suggested to have lower speciation and higher extinction rates than diploids (Mayrose et al., 2011). Consequently, Arrigo and Barker (2012) suggested that the high abundance of polyploids is a consequence of their high formation rate rather than of accelerated diversification of polyploids, but the inconsistent patterns of evolution following polyploidy make it challenging to develop a “unified theory” of polyploidy (Soltis et al., 2016).

The genus *Silene* L. (Caryophyllaceae) includes 800 to 900 mostly diploid (2*n* = 2*x* = 24) species distributed mainly across the Northern Hemisphere (Petri and Oxelman, 2011; Oxelman et al., 2013). Extensive polyploidisation has accompanied evolution of North American (Popp and Oxelman, 2007) and – to a lesser extent – arctic (Popp et al., 2005) and Asian (Petri and Oxelman, 2011) members of *Silene* sect. *Physolychnis* (Benth.) Bocquet, which includes diploids, tetraploids, hexaploids and octoploids (Petri and Oxelman, 2011; Rice et al., 2015). In North America most native species are tetraploids or octoploids (Popp and Oxelman, 2007), whereas little is known about the role of polyploidisation in the evolution of South American *Silene* sect. *Physolychnis.* The section includes species with upright stems, irregular thyrsoid inflorescences and mostly small, often dissected petal limbs (Petri and Oxelman, 2011). It was most thoroughly studied by Bocquet (1969), but based on molecular studies (e.g., Popp et al., 2005; Popp and Oxelman, 2007; Jenkins and Keller, 2010; Rautenberg et al., 2010; Petri and Oxelman, 2011) additional species were included in the section, thus establishing a morphologically rather diverse but phylogenetically well-supported monophyletic group (Petri and Oxelman, 2011).

For the Andes, the Andean Precordillera and other mountain areas (e.g., Sierra de Córdoba, Sierra de la Ventana) as well as for the Patagonian lowlands of South America, 17 species of *S.* sect. *Physolychnis* were recognized by Bocquet (1969) and two more by Pedersen (1984), namely *S. melanopotamica* Pedersen and *S. antarctica* (Kuntze) Pedersen; the latter was considered conspecific with *S. patagonica* (Speg.) Bocquet by Bocquet (1969). Bocquet (1969) classified the South American taxa into three subsections, the biggest being *S.* subsect. *Chilenses* Bocquet with ten species, distributed mostly in the Argentinian and Chilean Andes and the Patagonian steppes. *Silene* subsect. *Genovevanae* Bocquet includes five species distributed mostly in the northern parts of the Andes between northern Argentina and Chile and Colombia. The smallest subsection is *S.* subsect. *Songaricae* Bocquet with two species distributed between the provinces Jujuy and Mendoza in Argentina. Chromosome numbers were reported only for two species (Rice et al., 2015), tetraploid *S. andicola* Gill. (Bocquet and Favarger, 1971) and diploid *S. magellanica* (Desr.) Bocquet (Moore, 1981).

Many Northern American temperate plant lineages have colonized the high-elevation South American Andes since overland connections were established between the two continents (Raven, 1963; Bell and Donoghue, 2005; Hughes and Eastwood, 2006; Hughes et al., 2013; Bacon et al., 2015; Simpson et al., 2017). Previous analyses indicated a single migration from North to South America, but only three South American *Silene* species were studied. In internal transcribed spacer (ITS) and low-copy nuclear RPA2 and partly RPD2a phylogenetic trees the South American accessions were positioned in a clade with North American tetraploid *S. occidentalis* S. Watson and *S. verecunda* S. Watson with moderate support, whereas in other phylogenetic trees the relationship between North and South American taxa remained unresolved (Popp and Oxelman, 2007). In addition, South American *S. chubutensis* (Spegazz.) Bocquet and *S. thysanodes* Fenzl were positioned in the same clade with *S. occidentalis, S. polypetala* (Walter) Fernald & B. G. Schubert, *S. scouleri* Hook. and *S. verecunda* in a plastid DNA sequence tree (Petri and Oxelman, 2011). It remains unknown if other South American species had the same origin or originated from independent migrations from North to South America. It is also unclear, how the considerable morphological diversity of South American taxa reflects phylogenetic relationships within the group. Overall, the Andean high-elevation taxa mostly have large, spatulate to oblanceolate leaves, whereas low-elevation Patagonian taxa have narrower, linear to narrowly (ob)lanceolate leaves (Bocquet, 1969; Pedersen, 1984), but this might be a result of eco-morphological differentiation and/or evolutionary divergence.

To disentangle the origin and diversification of South American *Silene* sect. *Physolychnis* we use phylogenetic analyses of nuclear ITS and plastid *psbE*-*petG* and *matK* sequences, flow cytometric genome size estimations and chromosome counts, including 13 South American species. We address the following questions: (1) are the South American species of *Silene* sect. *Physolychnis* monophyletic, resulting from a single migration from North America?; (2) what role did polyploidisation play in the evolution of South American species and which lineages have been involved in their diversification?, and (3) is the morphological differentiation, especially the leaf shape, correlated to the evolutionary history or rather to divergent habitats?

## Materials and Methods

### Plant Material

**FIGURE 1:**
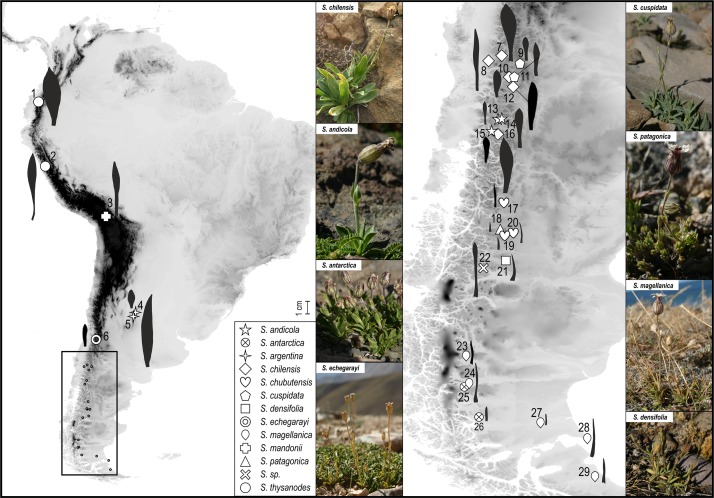
Sampled populations of *Silene* in South America, detailed information is given in Supplementary Table S1. The frame in the left map shows the position of the area depicted in the right map. Leaf shapes are depicted next to the sampling points. The photos represent some of the analyzed species in their natural habitats.

21 populations belonging to 11 species were collected in South America, mostly Argentina and Chile, between 2006 and 2008. In most cases silica-gel material and in all cases herbarium vouchers were collected, often with ripe seeds. Additionally, seven populations (2, 4, 5, 8, 13, 14, 22) belonging to five species were sampled from herbaria and 17 GenBank sequences from previous studies were added, thus resulting in a dataset of 29 populations belonging to 13 species (Figure 1 and Supplementary Table S1). The species were identified following Bocquet (1969) and Pedersen (1984); several accessions were collected at the localities listed by the two authors, thus facilitating the identification. Outgroup species, mostly from North America, were sampled from different herbaria and we also included 106 GenBank sequences from previous studies (Supplementary Table S2).

### Chromosome Numbers

Chromosome numbers were determined for six *Silene* species (Supplementary Table S1). Seeds were germinated on moist paper in Petri dishes and actively growing root tip meristems were collected and pre-treated with 0.002 M aqueous solution of 8-hydroxyquinoline for 2.5 h at room temperature and 2.5 h at 4°C, fixed in ethanol and glacial acetic acid (3 : 1), and stored at -20°C until use. Meristems were hydrolysed in 5N HCl for 20 min at room temperature, washed with tap water and stained with Schiff’s reagent for 1 h (Weiss-Schneeweiss et al., 2009). Chromosome spreads were prepared by squashing a stained meristem in a drop of acetic acid (60%) under the coverslip, and analyzed using an AxioImager M2 microscope (Carl Zeiss, Vienna, Austria). Images were acquired with a CCD camera and files processed using AxioVision ver. 4.8 (Carl Zeiss, Vienna, Austria) using only those functions that apply to the whole image equally.

### Relative Genome Size (RGS) Estimation With Flow Cytometry

Relative genome size was typically measured for four to six individuals per population of 18 populations representing nine species (Supplementary Table S1). Relative fluorescence intensity of silica-dry samples was estimated by DAPI flow cytometry according to the simplified one-step methodology using Otto buffers as detailed in Doležel et al. (2007). *Bellis perennis* (2C = 3.38 pg; Schönswetter et al., 2007) served as an internal reference standard. A Partec PA II cytometer equipped with a UV HBO lamp was used to record fluorescence values of 3000–5000 particles, depending on the sample quality. Due to the relatively frequent left-hand shoulder of the sample peak, manual gating was applied before the standard/sample peak ratio was calculated with FloMax software (Partec, Germany). Coefficients of variation of the G0/G1 peaks of the internal standard and the sample varied from 1.98 to 3.23% (mean 2.46%) and from 2.52 to 7.82% (mean 4.78%), respectively.

### DNA Extraction, PCR and Sequencing

Total genomic DNA was extracted from herbarium specimens or silica-gel dried material following the protocol described by Oxelman et al. (1997) and purified using the QIAquick purification kit protocol (QiaGen).

PCRs were performed as described by Frajman et al. (2009). PCR products were purified using Multiscreen PCR (Millipore) according to the manufacturer’s protocol and then sequenced with the PCR or nested primers (see Frajman et al., 2009) using the BigDye Terminator Cycle Sequencing Kit (Applied Biosystems), and visualized on ABI 3700 or ABI3730XL (Applied Biosystems). Sequencing reactions were performed by Macrogen Inc.

Contigs were assembled and edited using Geneious Pro 5.3.6 (Drummond et al., 2011). Base polymorphisms were coded using NC-IUPAC ambiguity codes as described by Frajman and Oxelman (2007). GenBank numbers of ITS sequences are presented in Supplementary Tables S1, S2.

### Phylogenetic Analyses

South American *Silene* accessions were first aligned to all Sileneae sequences in BoxTax database (Oxelman et al., 2013) available in 2016 using Geneious Pro 5.3.6 (Drummond et al., 2011) and the three datasets were analyzed using FastTree v.2.1.5 (Price et al., 2010) with the GTR model and Gamma20 likelihoods. Based on the inferred trees (not shown) as well as the study of Popp and Oxelman (2007) all South American accessions, their most closely related species and some additional taxa representing different groups of *Silene* sect. *Physolychnis* were sampled (see Supplementary Table S1) and their alignments analyzed as described below. Plastid *matK* and *psbE*-*petG* sequences were concatenated and analyzed together.

Maximum parsimony (MP) and MP bootstrap (MPB) analyses for ITS and plastid datasets were performed using PAUP 4.0b10 (Swofford, 2002). The most parsimonious trees were searched for heuristically with 100 replicates of random sequence addition, TBR swapping, and MulTrees off. The swapping was performed on a maximum of 1000 trees (nchuck = 1000). All characters were equally weighted and unordered. The data set was bootstrapped using full heuristics, 1000 replicates, TBR branch swapping, MulTrees option off, and random addition sequence with five replicates. *Silene acaulis* L. and *S. menziesii* Hooker were used for rooting in the plastid and ITS datasets, respectively, based on Popp and Oxelman (2007) and preliminary analyses described above.

Bayesian analyses were performed with MrBayes 3.2.1 (Ronquist et al., 2012) applying the SYM+Γ (ITS) and GTR+Γ (plastid dataset) substitution models proposed by the Akaike information criterion implemented in MrAIC.pl 1.4 (Nylander, 2004); *matK* and *psbE*-*petG* alignments were analysed separately with MrAIC but for both the same substitution model was proposed. Values for all parameters, such as the shape of the gamma distribution, were estimated during MrBayes analyses. The settings for the Metropolis-coupled Markov chain Monte Carlo process included four runs with four chains each (three heated ones using the default heating scheme), run simultaneously for 10,000,000 generations each, sampling trees every 1,000th generation using default priors. The posterior probability (PP) of the phylogeny and its branches was determined from the combined set of trees, discarding the first 1001 trees of each run as burn-in.

ITS and plastid datasets were pruned for divergence times estimation. Several distantly related outgroup taxa and some accessions of closely related species for which several accessions were available were removed. The resulting matrices thus included the same species. The analyses were performed using BEAST ver. 1.8.2 (Drummond et al., 2012). Prior to analyses, we excluded all longer (>5 bp) autapomorphic insertions from the plastid alignment. Birth-death speciation prior with incomplete sampling (Stadler, 2009) and GTR+Γ substitution model with estimated base frequencies were used for phylogeny inference. A lognormal relaxed clock with a weakly informative prior on the clock rate (exponential with mean 0.001) was applied. The prior age of the root was set to 3.37 million years with a normally distributed standard deviation of 0.9, which corresponds to the median age and 95% highest posterior densities (HPD) interval of the corresponding node obtained from the dating analysis by Sloan et al. (2009). In that study South *American S. argentina* (Pax) Bocquet was positioned in a polytomy with other species of *S*. sect. *Physolychnis* and the divergence of the clade was dated to 3.37 Ma (HPD, 1.69–5.21 Ma). The analyses were run for 10 million generations, logging parameters every 1000 generations. The performance of the analyses was checked in Tracer 1.6.0 (Rambaut et al., 2014); both the effective sample sizes and mixing were appropriate (i.e., ESS always exceeded 100 and the parameter traces showed the chain fluctuating around the equilibrium). The maximum clade credibility trees (MCC) showing the mean ages were produced and annotated with Tree Annotator (part of the BEAST package) after removing burnin and visualized with FigTree 1.4.2 (Rambaut, 2014).

### Leaf Shape Analyses

To compare the leaf shapes among populations of different species we plotted the scanned leaf shapes of the basal leaves on Figure 1. Additionally, we measured (1) the leaf length, (2) the maximal leaf width as well as (3) the distance from the leaf basis to the point of maximal leaf width for all sampled populations of subsect. *Chilenses*. We also calculated the ratios between 1 and 2, and 3 and 1, to describe the leaf shape.

## Results

### Chromosome Numbers and Relative Genome Size

**FIGURE 2:**
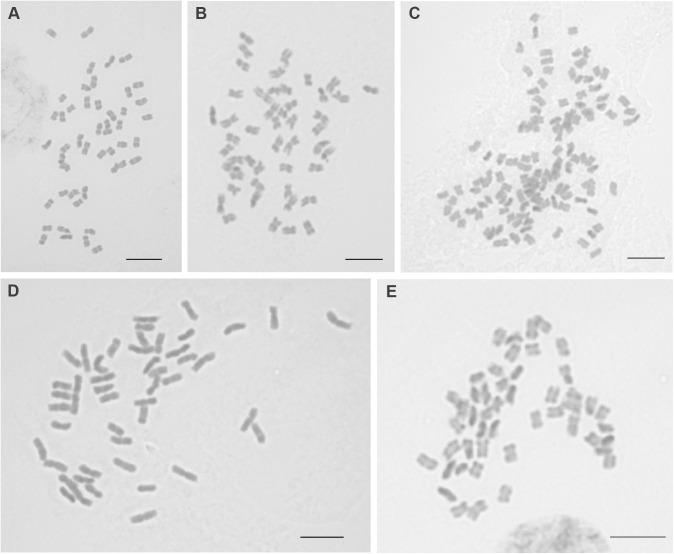
Mitotic chromosomes of analyzed *Silene* species. **A**, *Silene antarctica* (26) 2*n* = 4*x* = 48; **B**, *S. chubutensis* (20) 2*n* = 4*x* = 48; **C**, *S. echegarayi* (6) 2*n* = 10*x* = 120; **D**, *S. magellanica* (24) 2*n* = 4*x* = 48; **E**, *S. patagonica* (18) 2*n* = 4*x* = 48. Scale bar 5 μm. Population numbers in parentheses correspond to Supplementary Table S1 and Figure 1.

Chromosome numbers were established for 12 populations representing six *Silene* species (Supplementary Table S1 and Figure 2). The basic chromosome number of all individuals was inferred to be *x* = 12. Two ploidy levels were recorded, i.e., tetraploid (2*n* = 4*x* = 48) and decaploid (2*n* = 10*x* = 120). Each species, regardless of the number of individuals analyzed, represented only one ploidy level. Four species were tetraploid [*S. antarctica, S. chubutensis* (Spegazz.) Bocquet, *S. magellanica, S. patagonica*] and two decaploid, although establishing unambiguously exact chromosome numbers for those two individuals was no trivial task [*S. chilensis* (Naudin) Bocquet, *S. echegarayi* (Hieron.) Bocquet]. The karyotypes cannot be directly compared without additional markers, as it is impossible to define homo- or homeologous chromosome pairs based on chromosome morphology alone, especially given their very similar sizes and types (mostly metacentrics and submetacentrics with some acrocentrics). The average sizes of the chromosomes were similar within individual species, but differed among the species with *S. magellanica* having chromosomes larger than other analyzed taxa (Figure 2).

**FIGURE 3:**
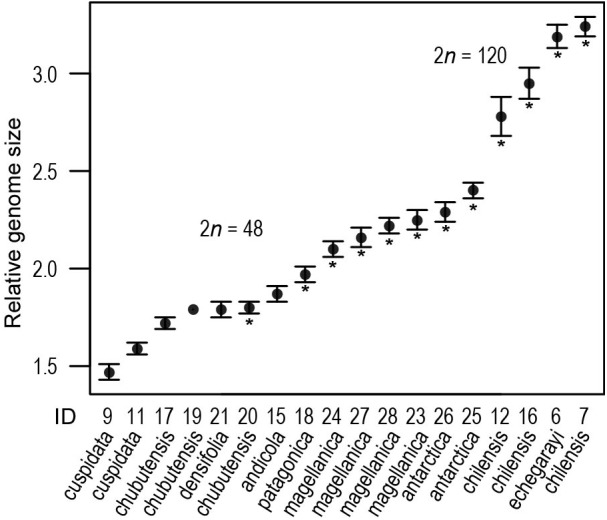
Relative genome size variation in South American *Silene* (see also Supplementary Table S1). Population numbers correspond to Supplementary Table S1 and Figure 1. Shown are population mean values (dot) and standard deviation, asterisks indicate the populations for which the chromosome number was estimated.

According to RGS the inference of ploidy-levels was not clear-cut (Figure 3), but available chromosome counts and RGS for some populations allowed the inference of DNA-tetraploidy in populations for which chromosome numbers were not estimated, at least for two populations of *S. chubutensis*, for which one count from a third population was available and for *S. densifolia*, which had the RGS very similar to that of *S. chubutensis* (ID 20), for which the chromosome number was established (Supplementary Table S1). The RGS of tetraploids ranged from 1.47 in *S. cuspidata* (ID 9) to 2.40 in *S. antarctica* (25), and in decaploids from 2.78 to 3.24, thus exhibiting 2.2-fold variation; both latter values are from *S. chilensis* (12, 7). Also among different populations of some species the RGS varied considerably (Supplementary Table S1 and Figure 3).

### Plastid and ITS Sequences

**FIGURE 4:**
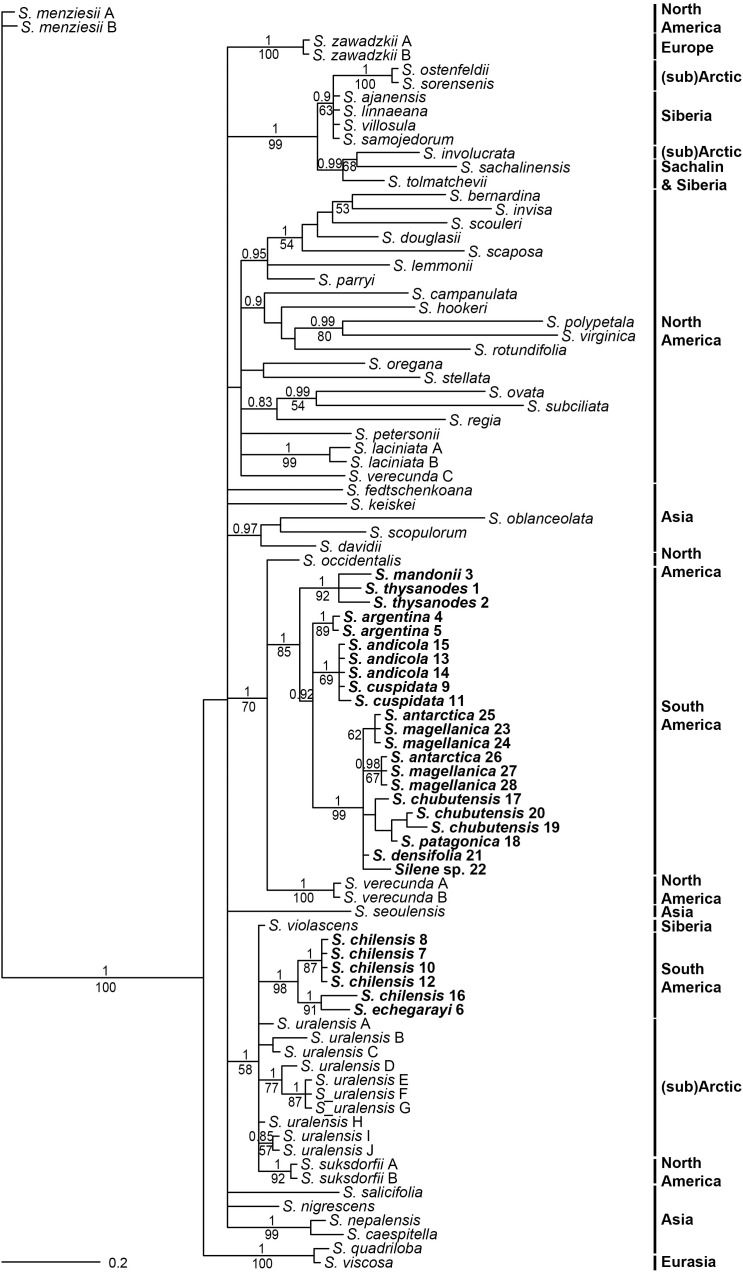
Bayesian consensus phylogram inferred by ITS sequences. Population identifiers correspond to Supplementary Tables S1, S2 and Figure 1. Numbers above branches are posterior probabilities >0.80, those below branches maximum parsimony bootstrap values >50%. Geographic distribution of species in indicated in bold.

Internal transcribed spacer sequences of South American accessions were between 746 (*Silene* sp. 22) and 748 (*S. chilensis* 7, 10, 12) bp long. Topologies inferred by parsimony and Bayesian analyses were congruent, as no strong incongruences (different strongly supported clades with PP > 0.95 and/or BS > 70%) were detected. Parsimony analysis inferred a lower number of clades, which mostly had lower support compared to Bayesian inference (Figure 4). South American *Silene* species were positioned in two monophyletic, but clearly divergent clades. One consisted of *S. chilensis* and *S. echegarayi* (posterior probability, PP 1, maximum parsimony bootstrap, BS 98%) and was positioned in a clade (PP 1, BS 58%) with *S. suksdorfii* B. L. Robinson, *S. uralensis* (Rupr.) Bocquet and *S. violascens* (Tolm.) V. V. Petrovsky & Elven. All other South American species were in a clade (PP 1, BS 85%) in trichotomy (PP 1, BS 70%) with North American *S. occidentalis* and *S. verecunda*.

**FIGURE 5:**
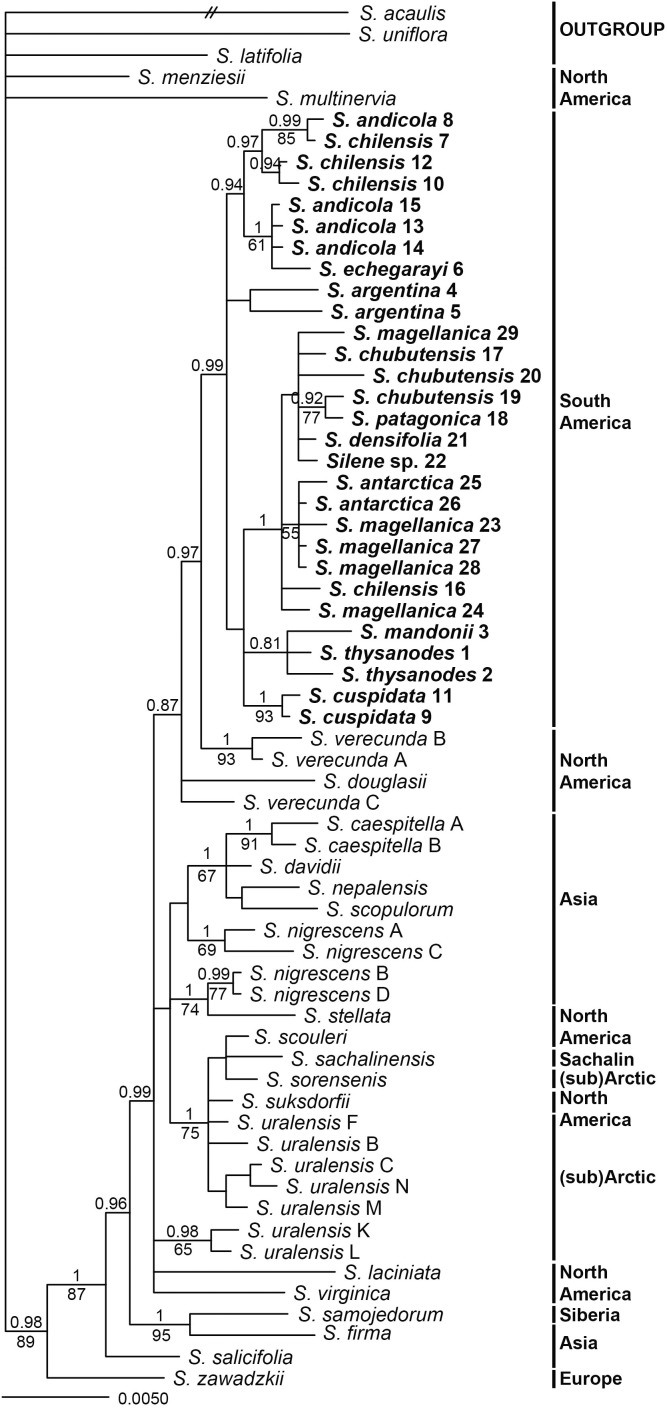
Bayesian consensus phylogram inferred by plastid (concatenated *matK* and *psbE*–*petG*) sequences. Population identifiers correspond to Supplementary Tables S1, S2 and Figure 1. Numbers above branches are posterior probabilities >0.80, those below branches maximum parsimony bootstrap values >50%. Geographic distribution of species in indicated in bold.

All *matK* sequences of South American accessions were 869 bp long, those of *psbE*–*petG* varied in length between 1437 bp (*S. densifolia* (Dusen) Bocquet 21) and 1487 bp (*S. chilensis* 7). Topologies inferred by parsimony and Bayesian analyses of the concatenated plastid dataset were largely congruent, as no strong incongruences (different strongly supported clades with PP > 0.95 and/or BS > 70%) were detected. Parsimony analysis inferred a lower number of clades, which mostly had lower support compared to Bayesian inference (Figure 5). South American *Silene* species formed a monophyletic group (P 0.99) sister to two accessions of North American *S. verecunda* (PP 0.97). All other outgroup species formed several clades with different support values. One of them, clearly separated from South American accessions, consisted of *S. sachalinensis* F. Schmidt, *S. scouleri, S. sorensenis* (B. Boivin) Bocquet, *S. suksdorfii* and *S. uralensis* (PP 1, BS 75%).

**FIGURE 6:**
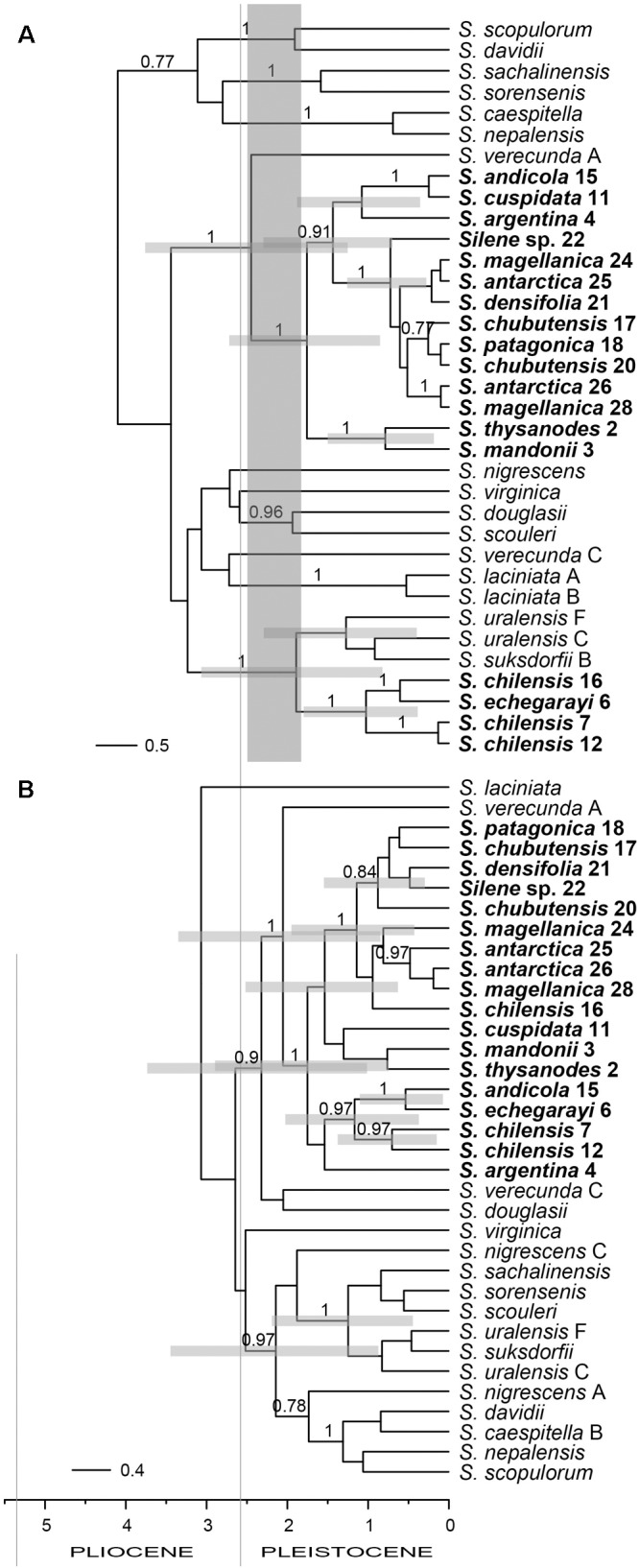
Bayesian consensus chronograms (Maximum Clade Credibility trees) showing the temporal diversification of South American *Silene* sect. *Physolychnis* based on ITS **(A)** and plastid *matK* and *psbE*–*petG* sequences **(B)**. Numbers above branches are PP values >0.75. Horizontal gray bars (depicted only for clades of interest) correspond to 95% highest posterior densities (HPD) of the age estimates. Population identifiers correspond to Supplementary Tables S1, S2 and Figure 1. The vertical bar in **A** shows the time lag between the migration of the South American lineage from North America and the putative hybridisation with the *S. uralensis* lineage.

The relationships in phylogenetic trees inferred by Bayesian analyses using BEAST were largely unresolved, resulting in a basal polytomy (Figure 6), in accordance with the results described above (Figures 4, 5). Only some clades had a support exceeding PP 0.95. In the ITS tree (Figure 6A) one of these clades included most of the South American accessions (PP 1) as sister (PP 1) to one accession of *S. verecunda*. The other such clade included *S. chilensis* and *S. echegarayi* (PP 1) as sister (PP 1) to *S. uralensis* and *S. suksdorfii*. In the plastid tree (Figure 6B) all South American species were in a clade (PP 1) as sister (PP 1) to one accession of *S. verecunda*, whereas *S. uralensis* and *S. suksdorfii* were in a clade (pp 0.97) with *S. nigrescens* and some other species. The divergence of South American species from North American *S. verecunda* was dated to the late Pliocene to early Pleistocene, 2.45 Ma (HPD, 1.25–3.76 Ma) in the ITS tree and to 2.05 Ma (HPD, 0.85–3.35 Ma) in the plastid tree. *Silene chilensis* and *S. echegarayi* diverged from the *S. uralensis* alliance 1.89 Ma (HPD, 0.82–3.07 Ma).

**FIGURE 7:**
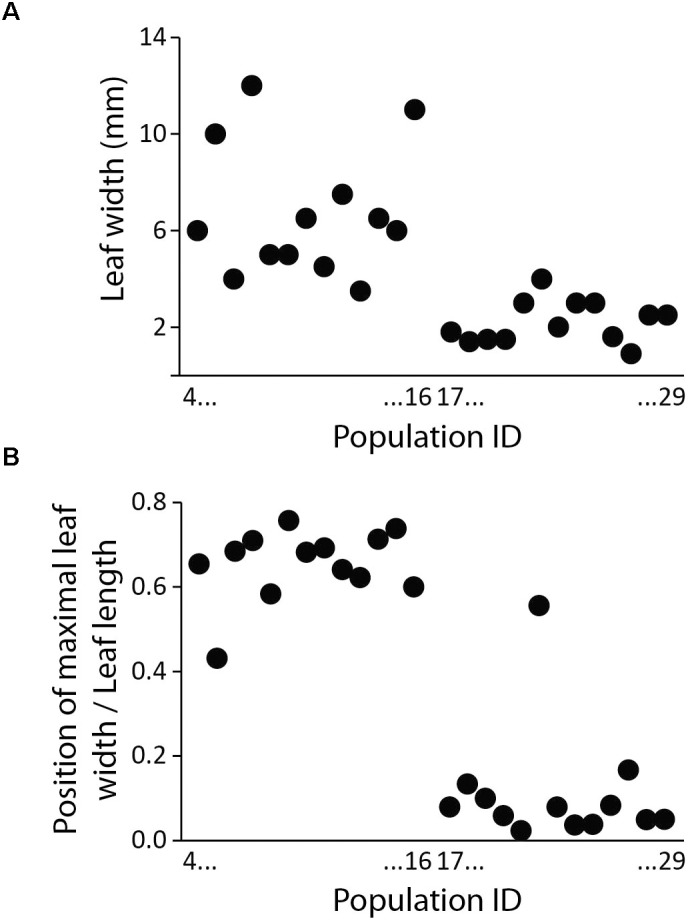
Leaf shape of populations 4 to 29 (correspond to Supplementary Table S1 and Figure 1) belonging to *Silene* subsect. *Chilenses*, expressed as leaf width **(A)** and distance from the leaf basis to the point of maximal leaf width **(B)** between the high-elevation taxa (populations 4–16) and the low-elevation taxa (populations 17–29). Populations are ordered from left to right according to increasing ID (i.e., following a North to South gradient).

### Leaf Shape Variation

As shown in Figure 1 leaf shape differs among high- and low-elevation populations. Within *S*. subsect. *Chilenses* the high-elevation taxa have broader, spatulate to oblanceolate leaves, which are widest in the upper half, whereas the low-elevation species have narrower, usually linear leaves, which are normally widest at the base (Supplementary Table S1 and Figures 1, 7).

## Discussion

### Single Migration From North to South America in *Silene* Sect. *Physolychnis*

Phylogenies inferred from nuclear ITS and plastid DNA sequences (Figures 4–6) suggest a single origin of South American species of *S.* sect. *Physolychnis*, followed by allopolyploidisation in the *S. chilensis* lineage. The datasets indicate that South America was colonized by a lineage sister to North American *S. verecunda* and *S. occidentalis*. *Silene verecunda* is a tetraploid, morphologically and ecologically exceptionally variable species occurring in the western United States from 0 to 3400 m (Morton, 2005). Morphologically it is fairly similar to the South American species, even if many species of *Silene* sect. *Physolychnis* resemble in habit. In contrast, *S. occidentalis*, which is endemic to California, differs considerably in morphology from South American species and *S. verecunda*, especially in having a remarkably prolonged tubular calyx and deeply lobed pink petals (Morton, 2005). Many Northern American temperate plant lineages have colonized the South American Andes after overland connections were established between the two continents, but the timing of migrations differed among the groups (Bell and Donoghue, 2005; Hughes and Eastwood, 2006; Popp et al., 2011; Hughes et al., 2013; Bacon et al., 2015; Simpson et al., 2017). Previous studies have suggested the full closure of the Isthmus of Panama, triggering the Great American Biotic Interchange (GABI), to have happened 3.5 Ma (Duque-Caro, 1990; Haug and Tiedemann, 1998; Coates et al., 2004; Coates and Stallard, 2013), but Bacon et al. (2015) demonstrated significant waves of dispersal of terrestrial organisms between both continents also at earlier times. The present day elevations of the northern and northern-central Andes were reached during or soon after the late Miocene, which allowed them to serve as a colonization corridor during the GABI and created habitats suitable for mountain plants in the late Pliocene (Burnham and Graham, 1999; Gregory-Wodzicki, 2000; Hughes and Eastwood, 2006; Mora et al., 2009; Patterson and Costa, 2012).

In a dated plastid *matK* phylogeny of *Silene* (Sloan et al., 2009) South American *S. argentina* was positioned in a polytomy with other, also North American species of *S.* sect. *Physolychnis* and the divergence of the clade was dated to 3.37 Ma (HPD, 1.69–5.21 Ma). Using this date as secondary calibration, we inferred the migration of *S.* sect. *Physolychnis* from North to South America. The divergence times inferred by ITS and plastid data differ slightly, but the HPD intervals overlap largely, i.e., 2.45 Ma (HPD, 1.25–3.76 Ma) in the ITS tree and 2.05 Ma (HPD, 0.85–3.35 Ma) in the plastid tree (Figure 6). The migration thus likely happened in the late Pliocene or early Pleistocene, i.e., after the final closure of the Isthmus of Panama and the final uplift of the northern Andes (Gregory-Wodzicki, 2000). This is in line with previous evidence that the upland Andean habitats have been available for plant colonization only since the late Pliocene and early Pleistocene 2 to 4 Ma and several plant groups, including *Silene* sect. *Physolychnis*, diversified in this area in the Pleistocene (Burnham and Graham, 1999; Gregory-Wodzicki, 2000; Hughes and Eastwood, 2006; Popp et al., 2011; Madriñán et al., 2013; Hughes and Atchison, 2015). In several genera like *Astragalus* (Fabaceae; Scherson et al., 2008), *Calceolaria* (Calceolariaceae; Cosacov et al., 2009) *Gentianella* (Gentianaceae; Von Hagen and Kadereit, 2001), *Hypericum* (Hypericaceae; Nürk et al., 2014), *Lupinus* (Fabaceae; Hughes and Eastwood, 2006), and *Valeriana* (Valerianaceae; Bell and Donoghue, 2005) rapid diversifications leading to high numbers of species in the Andes have been evidenced, but in *Silene* sect. *Physolychnis* the number of species remained relatively low.

### Allopolyploidisation and Origin of Decaploid Species

Most of the South American species analyzed here are tetraploid (Figures 2, 3 and Supplementary Table S1), similar to their North American sister taxon *S. verecunda* (Morton, 2005). A decaploid chromosome number (2*n* = 10*x* = 120) established for the high Andean species *S. chilensis* and *S. echegarayi* (Figure 2 and Supplementary Table S1) is the highest ploidy level ever reported in *Silene* sect. *Physolychnis*, since only octoploids were previously known (Popp and Oxelman, 2007; Petri and Oxelman, 2011; Rice et al., 2015); up to 240 chromosomes have been reported for European *S. ciliata* Pourr. (Blackburn, 1933). The diploid chromosome count for *S. magellanica* by Moore (1981) is probably erroneous, as no diploids were established by our chromosome counts neither for this nor any other South American species belonging to *S.* sect. *Physolychnis*.

A discordant position of decaploid South American species in ITS and plastid trees (Figures 4–6) suggests their allopolyploid origin. Decaploid species are positioned in the same clade with tetraploid South American species in the plastid tree (Figure 5), where most accessions (with exception of population 16 of *S. chilensis*) appear in the same clade with morphologically very similar and sympatric *S. andicola*; thus, the latter likely served as a maternal parent in the allopolyploid origin of the decaploids. On the other hand, in the ITS tree decaploids are positioned in a clade with the diploid arctic/subarctic *S. uralensis*, the North American octoploid *S. suksdorfii* and the North Asian *S. violascens*. Nowadays none of these or closely related species occur in South America and it remains unclear how the hybridisation has happened. In any case, our dating analyses indicate a time lag between the migration to South America and the putative hybridisation with the *S. uralensis* lineage resulting in the allopolyploidisation as the divergence of decaploids from the *S. uralensis* lineage was dated to 1.89 Ma (HPD, 0.82–3.07 Ma).

Previous research has shown that diploid *S. uralensis* was involved also in the alloploid origin of tetraploid subarctic/arctic *S. involucrata* (Cham. & Schltdl.) Bocquet, but as a maternal lineage (Popp et al., 2005). In this case the distribution range of the paternal Siberian/northeastern Asian *S. ajanensis* (Regel.) Vorosch. lineage nowadays overlaps with that of *S. involucrata* (Popp et al., 2005). In combination with plastid DNA sequences, ITS has often been used to infer origins of polyploid plants (Sang et al., 1995; Ge et al., 1999; Popp et al., 2005; Popp and Oxelman, 2007; Kuzmanović et al., 2017). However, since the homoeologous ITS repeats in an allopolyploid often are homogenized toward one of the parental types by concerted evolution or chimeric sequences are formed (Wendel et al., 1995), the inference of allopolyploid origin is only possible if the homogenization is directed toward the paternal lineage (Smedmark and Eriksson, 2002; Popp et al., 2005), which was also the case in South American *Silene* sect. *Physolychnis*. The homogenisation seems to be complete, as we observed only two polymorphic sites in all ITS accessions of decaploid *Silene*.

### Morphological Differentiation in South American *Silene* Sect. *Physolychnis*

The South American species of *Silene* sect. *Physolychnis* exhibit a great variability in habitats as well as in morphology. Most of the species occur at higher altitudes of the Andes, the Andean Precordillera and other mountain areas in Argentina (e.g., Sierra de Córdoba, Sierra de la Ventana), but some inhabit also Patagonian dry steppes and semi-deserts (Bocquet, 1969; Pedersen, 1984). Based on the calyx characters, Bocquet (1969) divided South American species into three subsections. The first, *S*. subsect. *Genovevanae* Bocquet includes five species from the northern Andes from Venezuela to northern Argentina. From this subsection we included *S. mandonii* (Rohrb.) Bocquet and *S. thysanodes* Fenzl in our phylogenetic analyses; in the ITS tree (Figure 4) they form a well-supported clade (PP 1, BS 92%) sister to all other taxa, which were by Bocquet (1969) classified in *S.* subsect. *Chilenses*. In the plastid tree the relationships between the two subsections are less clear, as several clades, some with low support, form a polytomy (Figure 5). The two species (*S. argentinensis* Hauman and *S. bersieri* Bocquet) from the third subsection, *S.* subsect. *Songaricae* Bocquet were not included in our analyses.

Within *S.* subsect. *Chilenses* we observed a great variability in leaf shapes. Whereas the high-elevation taxa – similarly as those from subsect *Genovevanae* – mostly have broad, spatulate to oblanceolate leaves, which are widest in the upper half, the low-elevation species have narrow, usually linear leaves, which are normally widest at the base (Supplementary Table S1 and Figures 1, 7). Such a shift in the leaf shape is likely an adaptation to the dry and continental environments of the Patagonian steppes, as leaf size strongly correlates with climate as shown for different plant groups (e.g., Xu et al., 2009; Guerin et al., 2012). Phylogenetic patterns are congruent with this eco-morphological separation as well. High-elevation broad-leaved Andean *S. andicola* and *S. cuspidata* are included in a clade with good support (PP 1, BS 69%) in the ITS tree; in the plastid tree the former is in a poorly supported clade (PP 0.94) with – also broad-leaved – *S. chilensis* and *S. echegarayi* and the latter forms its own clade (PP 1, BS 93%). Another broad-leaved clade includes the geographically more distant *S. argentina* from the Sierra de Córdoba in Argentina (ITS: PP 1, BS 89%; plastid tree: no support), which occurs also in the Sierra de San Luis and the Sierra de la Ventana (Bocquet, 1969). The third clade includes narrow-leaved Patagonian steppe species (ITS: PP 1, BS 99%; plastid tree: PP 1, including also accession 16 of *S. chilensis*), which are morphologically very variable, also in leaf size, having very short to long leaves. The voucher of population 22 had a rather unusual leaf shape for this group, having slightly spathulate leaves, which are broadest in the upper third (Figure 1); it was not possible to identify it as any of the described species. We acknowledge that also in some other cases the identification of narrowed-leaved Patagonian steppe species was not straight forward, neither by applying the keys and descriptions by Bocquet (1969), nor those by Pedersen (1984). Also the lack of resolution among these species in the phylogenetic trees suggests that a comprehensive taxonomic revision of this group is needed and that some species might merely be ecotypes adapted to specific habitats (e.g., exposed screes or rock crevices).

## Conclusion

Our study provides new insights into origin and diversification of *Silene* sect. *Physolychnis* from South America. The analyzed nuclear ITS and plastid sequences indicate a single migration of the *S. verecunda* lineage from North to South America and subsequent allopolyploidisation with the *S. uralensis* lineage. To corroborate this hypothesis further phylogenetic studies including DNA sequences from additional nuclear and plastid regions are needed. Moreover, sampling of South American species not included in our study is needed to corroborate a single origin of all South American members of this section. Finally, low phylogenetic resolution within different species groups and high intraspecific morphological variability calls for a taxonomic revision of South American *Silene* sect. *Physolychnis*.

## Author Contributions

BF designed the study, coordinated its implementation, conducted field work, performed karyological and phylogenetic analyses, and wrote major parts of the paper. PS conducted field work and improved a draft of the paper. HW-S coordinated and conducted karyological analyses, wrote corresponding parts of the paper and improved its draft. BO was involved in study design, performed the phylogenetic analysis of the complete Sileneae datasets, coordinated the sequencing of most of the sequences, and improved a draft of the paper.

## Supplementary Material

The Supplementary Material for this article can be found online at: https://www.frontiersin.org/articles/10.3389/fgene.2018.00639/full#supplementary-material

## Conflict of Interest Statement

The authors declare that the research was conducted in the absence of any commercial or financial relationships that could be construed as a potential conflict of interest.
